# A Comprehensive Review on Cardiovascular Complications of COVID-19: Unraveling the Link to Bacterial Endocarditis

**DOI:** 10.7759/cureus.44019

**Published:** 2023-08-24

**Authors:** Anurag Bele, Vasant Wagh, Pratiksha K Munjewar

**Affiliations:** 1 Internal Medicine, Jawaharlal Nehru Medical College, Datta Meghe Institute of Higher Education & Research, Wardha, IND; 2 Community Medicine, Jawaharlal Nehru Medical College, Datta Meghe Institute of Higher Education & Research, Wardha, IND; 3 Medical Surgical Nursing, Smt. Radhikabai Meghe Memorial College of Nursing, Datta Meghe Institute of Higher Education and Research, Wardha, IND

**Keywords:** clinical management, mechanisms, diagnosis, immune response, pathogenesis, cardiovascular complications, bacterial endocarditis, covid-19

## Abstract

The global pandemic caused by severe acute respiratory syndrome coronavirus 2 (SARS-CoV-2) has ushered in a new era of understanding the multifaceted nature of infectious diseases. Beyond its well-documented respiratory impact, COVID-19 has unveiled intricate interactions with the cardiovascular system, with potential implications that extend to bacterial endocarditis. This review explores the complex interplay between COVID-19 and bacterial endocarditis, elucidating shared risk factors, theoretical mechanisms, and clinical implications. We examine the diverse cardiovascular manifestations of COVID-19, ranging from myocarditis and thromboembolic events to arrhythmias, and delve into the pathogenesis, clinical features, and diagnostic challenges of bacterial endocarditis. By analyzing potential connections, such as viral-induced endothelial disruption and immune modulation, we shed light on the plausible relationship between COVID-19 and bacterial endocarditis. Our synthesis highlights the significance of accurate diagnosis, optimal management, and interdisciplinary collaboration in addressing the challenges posed by these intricate interactions. In addition, we underscore the importance of future research, emphasizing prospective studies on bacterial endocarditis incidence and investigations into the long-term cardiovascular effects of COVID-19. As the boundaries of infectious diseases and cardiovascular complications converge, this review calls for continued research, vigilance, and coordinated efforts to enhance patient care and public health strategies in a rapidly evolving landscape.

## Introduction and background

The ongoing global pandemic caused by the severe acute respiratory syndrome coronavirus 2 (SARS-CoV-2) has brought to light a multitude of complex and diverse clinical manifestations. Initially characterized primarily as a respiratory illness, COVID-19 has demonstrated its propensity to affect multiple organ systems, including the cardiovascular system. The interconnectedness between viral infections and cardiovascular complications has been recognized. Nonetheless, the unprecedented scale and clinical impact of COVID-19 have intensified the need to unravel its intricate associations, particularly regarding bacterial endocarditis [1-4].

COVID-19, caused by the novel coronavirus SARS-CoV-2, was first identified in late 2019 and rapidly spread across the globe, leading to a profound global health crisis. The virus primarily targets the respiratory system, causing a range of symptoms from a mild flu-like illness to severe acute respiratory distress syndrome (ARDS). However, as the pandemic evolved, it became evident that COVID-19 can elicit diverse clinical manifestations beyond the respiratory tract, including neurological, gastrointestinal, and cardiovascular complications. This expanded understanding of COVID-19 prompted further investigation into the intricate interplay between the virus and various organ systems [5].

While the respiratory impact of COVID-19 garnered significant attention during the early stages of the pandemic, emerging clinical observations and research studies have underscored the substantial cardiovascular implications of the disease. Cardiovascular complications of COVID-19 encompass a spectrum of disorders, ranging from myocardial injury, arrhythmias, and myocarditis to thrombotic events and endothelial dysfunction. These complications have contributed to the morbidity and mortality associated with COVID-19 and raised critical questions about the underlying mechanisms driving cardiovascular involvement [6,7].

Bacterial endocarditis, characterized by inflammation of the endocardium and heart valve structures, has long been recognized as a serious and potentially life-threatening infection. It is typically caused by bacterial pathogens that access the bloodstream and adhere to damaged cardiac valves or endocardial surfaces. The resultant formation of microbial biofilms can lead to valvular destruction, heart failure, septic emboli, and other severe complications. The clinical course of bacterial endocarditis is complex, requiring prompt diagnosis and specialized treatment approaches. Given its potential for grave outcomes, exploring any potential link between COVID-19 and bacterial endocarditis holds clinical and research significance [8].

This review article aims to comprehensively examine the emerging understanding of the cardiovascular complications associated with COVID-19, focusing on the potential interplay between COVID-19 and bacterial endocarditis. By synthesizing existing literature and highlighting key clinical observations, this review aims to elucidate whether there exists a plausible connection between these two seemingly disparate entities. This article also seeks to identify shared risk factors, potential mechanistic pathways, and clinical implications while underscoring the importance of accurate diagnosis, management, and future research endeavors in this evolving landscape.

## Review

COVID-19 and cardiovascular complications

Overview of Cardiovascular Manifestations in COVID-19

Initially characterized as primarily affecting the respiratory system, COVID-19 has emerged as a condition with diverse and intricate cardiovascular implications, adding a layer of complexity to its clinical presentation. This expanded understanding has unveiled a range of cardiovascular complications that extend beyond the lungs. In this section, we provide a comprehensive overview of the key cardiovascular manifestations associated with COVID-19, highlighting their clinical significance and potential impact [9,10].

Myocarditis and pericarditis: Myocarditis, characterized by heart muscle inflammation and pericarditis, involving inflammation of the pericardium, have been observed among COVID-19 patients. These inflammatory processes within the cardiac structures can precipitate symptoms, such as chest pain, palpitations, and shortness of breath. In severe cases, they may lead to the development of arrhythmias and even heart failure. While the precise mechanisms driving COVID-19-associated myocardial and pericardial inflammation are still under investigation, it is believed that a combination of viral-induced damage, immune responses, and direct endothelial involvement contributes to these pathological changes [11].

Thromboembolic events: COVID-19 has been closely linked to an elevated risk of thrombotic events, including the formation of blood clots in deep veins (deep vein thrombosis), the migration of clots to the lungs (pulmonary embolism), and clot formation within arteries (arterial thrombosis). The underlying mechanisms driving this heightened thrombotic tendency involve a combination of endothelial dysfunction, widespread inflammation, and a hypercoagulable state triggered by the virus. These thrombotic complications contribute to the severity of the disease and pose challenges in managing COVID-19 patients [12].

Arrhythmias: The spectrum of arrhythmias seen in COVID-19 patients encompasses a wide range, from atrial fibrillation to more severe ventricular arrhythmias. The inflammatory milieu induced by the virus, combined with myocardial injury and electrolyte imbalances, can disrupt the normal electrical signalling within the heart, leading to irregular heart rhythms. These arrhythmias impact the patient's immediate clinical status and can have far-reaching implications on outcomes, necessitating careful monitoring and targeted interventions [13,14].

Recognizing these diverse cardiovascular complications underscores the need for a holistic approach to managing COVID-19 patients. Beyond its respiratory effects, the virus's intricate interactions with the cardiovascular system necessitate comprehensive evaluation, monitoring, and therapeutic strategies to address the multifaceted clinical challenges posed by COVID-19 [15].

Mechanisms underlying cardiovascular involvement

Understanding the intricate mechanisms by which SARS-CoV-2 affects the cardiovascular system is paramount for unravelling the pathophysiology of COVID-19-associated cardiovascular complications. The multifaceted nature of these complications emerges from the convergence of several interconnected mechanisms, each contributing to the observed cardiovascular involvement [16,17]:

ACE2 Receptors and Viral Entry

The angiotensin-converting enzyme 2 (ACE2) receptor is pivotal in facilitating SARS-CoV-2's entry into host cells. Notably, ACE2 receptors are prominently expressed on both cardiac and endothelial cells, rendering them potential sites for viral attachment and invasion. Upon viral entry, the intricate orchestration of cellular processes can result in direct myocardial injury and instigate endothelial dysfunction. This endothelial barrier disruption can subsequently compromise the structural and functional integrity of the cardiovascular system [18-20].

Immune Response and Cytokine Storm

Characterized by immune dysregulation, COVID-19 triggers a cascade of immune responses, sometimes culminating in a cytokine storm. This storm involves the uncontrolled release of pro-inflammatory cytokines, leading to widespread inflammation and tissue damage. The ensuing systemic inflammatory milieu poses a significant threat to myocardial and endothelial health, precipitating further damage and exacerbating cardiovascular complications [21-24].

Endothelial Dysfunction and Vascular Effects

Direct infection of endothelial cells by SARS-CoV-2 contributes to the emergence of endothelial dysfunction, a hallmark of COVID-19 pathogenesis. This dysfunction disrupts the finely-tuned equilibrium between vasodilation and vasoconstriction, thus impeding blood flow regulation. The consequences extend beyond impaired flow dynamics, as it fosters an environment conducive to microvascular thrombosis, inflammation within the vascular wall, and systemic vascular instability [25-29].

The intricate interplay among these mechanisms forms the foundation of the multifaceted landscape of COVID-19-associated cardiovascular complications. While each mechanism independently influences specific facets of cardiovascular involvement, their convergence often results in synergistic effects, amplifying the overall impact on the cardiovascular system. Grasping the intricate relationships between viral infection and cardiovascular consequences provides insight into the disease's complexity. It underscores the importance of holistic approaches in managing and mitigating the cardiovascular challenges posed by COVID-19 [30,31].

Bacterial endocarditis: pathogenesis and clinical features

Introduction to Bacterial Endocarditis

Bacterial endocarditis is a severe infectious condition characterized by the colonization and growth of microbial pathogens on the endocardium, heart valves, or other cardiac structures. While bacterial endocarditis has decreased over the years due to advances in medical care and prophylactic measures, it remains a critical clinical concern due to its potential for severe complications, including valvular destruction, heart failure, and embolic events. This section introduces the fundamental aspects of bacterial endocarditis, emphasizing its clinical significance and potential relevance in COVID-19 [32].

Pathophysiology of Infective Endocarditis

Infective endocarditis, a severe and potentially life-threatening condition, emerges due to an intricate interplay between microbial pathogens and various host factors, often centred around compromised or damaged cardiac structures. The intricate pathophysiological underpinnings of this condition form a critical foundation for recognizing its potential relationship with COVID-19. Several vital factors collectively contribute to the development of infective endocarditis, shaping its complex clinical manifestation [33,34]:

Microbial factors: Bacterial pathogens, frequently belonging to the staphylococci and streptococci families, are pivotal in initiating infective endocarditis. These pathogens exploit the opportunity presented by damaged endocardial surfaces, adhering to them and subsequently instigating the formation of microbial biofilms. These biofilms create a shielded microenvironment that protects the bacteria, effectively evading the host's immune response and resisting antibiotic treatment. This protective niche facilitates the persistence of bacterial infection and enhances the potential for embolic dissemination, a hallmark feature of this condition [35,36].

Host factors: Various host-related factors heavily influence the development of infective endocarditis. Predisposing conditions, such as pre-existing valvular abnormalities, congenital heart disease, or a history of intravenous drug use, create a conducive environment for bacterial colonization. These conditions lead to localized abnormalities within the heart, providing attachment sites for bacterial adherence. Furthermore, situations involving turbulent blood flow, often encountered near valve deformities or in areas of anatomical turbulence, contribute to the heightened susceptibility of endocardial surfaces to bacterial colonization. The cumulative effect of these host factors increases the likelihood of successful bacterial attachment and subsequent formation of infective endocarditis [37].

Understanding this intricate interplay between microbial and host factors in the context of infective endocarditis not only enhances our comprehension of the condition but also paves the way for recognizing potential associations with other clinical entities, such as COVID-19. By exploring these factors in greater detail, researchers and clinicians can unravel the complex pathogenesis underlying infective endocarditis and its potential interactions with emerging infectious diseases, contributing to improved diagnosis, management, and patient outcomes [38].

Clinical presentation and diagnosis of bacterial endocarditis

The clinical presentation of bacterial endocarditis, a complex and potentially life-threatening condition, encompasses a broad spectrum of symptoms that often pose diagnostic challenges for healthcare practitioners. Achieving timely and accurate diagnosis is paramount, as it facilitates initiating appropriate and targeted management strategies. In this section, we navigate the nuances of bacterial endocarditis's clinical features, diagnostic criteria, and the techniques employed in its identification [33].

Modified Duke Criteria

As a cornerstone in the diagnostic landscape of infective endocarditis, the Modified Duke Criteria provides clinicians with a systematic and structured framework for diagnosing this intricate condition. These criteria consider a comprehensive array of factors, including clinical manifestations, microbiological evidence, and results from various imaging modalities. By classifying cases into distinct categories, i.e., definite, possible, or rejected, the Modified Duke Criteria empower clinicians to make well-informed diagnostic decisions. This classification system not only aids in establishing a more accurate diagnosis but also contributes to enhanced patient management and prognosis [39].

Imaging Techniques

Given the complex nature of bacterial endocarditis, accurate visualization of cardiac structures and abnormalities is pivotal for both diagnosis and subsequent management. A suite of imaging modalities, prominently echocardiography, offered in both transthoracic and transesophageal variations, plays a central role. Echocardiography is a versatile tool that visualizes vegetation, valvular irregularities, and broader cardiac functionality. These visual insights confirm the diagnosis and provide valuable guidance for treatment planning, enabling healthcare providers to tailor interventions to the patient's unique clinical presentation [40,41].

As we further explore the potential links between COVID-19 and bacterial endocarditis, the foundational knowledge amassed from understanding the pathogenesis, clinical nuances, and diagnostic methodologies of bacterial endocarditis is a crucial bedrock. This understanding sharpens our ability to decipher potential connections and equips us to investigate whether COVID-19 might influence this formidable cardiovascular infection's risk, manifestation, or outcomes. The convergence of these insights fuels our pursuit of deeper comprehension, enabling us to navigate the intricate web of interactions between viral infections and bacterial complications, ultimately contributing to a more comprehensive approach to patient care and public health strategies [42,43].

Potential interplay between COVID-19 and bacterial endocarditis

Shared Risk Factors and Predisposition

The potential interplay between COVID-19 and bacterial endocarditis is a complex dynamic shaped by shared risk factors and underlying conditions that can create a fertile environment for both viral infection and bacterial colonization to occur. Gaining a deeper understanding of these factors can illuminate the intricate relationship between the two entities [44].

Immune Compromise

COVID-19 induces immune dysregulation, compromising the body's immune responses. Prolonged viral presence and the occurrence of cytokine storms, where the immune system releases excessive inflammatory molecules, could collectively weaken the immune defences. This susceptibility may provide an opportunistic window for secondary infections to take hold, including bacterial endocarditis. As the immune system becomes preoccupied with combating the viral invader, it may be less effective in recognising and responding to bacterial pathogens, potentially allowing for their colonization and the development of endocarditis [45].

Endothelial Damage

COVID-19 and bacterial endocarditis can damage the delicate endothelial layer lining blood vessels and cardiac structures. The resulting disruption of the protective endothelial barrier has far-reaching consequences. In the context of COVID-19, compromised endothelial integrity can facilitate the entry and replication of the virus within the cardiovascular system. Simultaneously, this endothelial damage may create sites of vulnerability where bacterial pathogens can adhere to and establish infections. The endothelial barrier disruption can be a double-edged sword, exacerbating the risk of viral and bacterial intrusions into cardiac tissues [46,47].

This intricate interplay between immune compromise and endothelial damage underpins the potential synergy between COVID-19 and bacterial endocarditis. The shared mechanisms of immune dysregulation and endothelial dysfunction underscore the relationship's complexity and emphasize the importance of interdisciplinary research efforts. Understanding these interconnected factors can pave the way for targeted interventions and improved patient care, preventing COVID-19-associated cardiovascular complications and managing the potential risk of bacterial endocarditis in affected individuals [48].

Case Studies and Reported Instances

Case studies and reported instances documented in the medical literature serve as illuminating windows into the potential connection between COVID-19 and bacterial endocarditis. These individual clinical accounts offer valuable insights and anecdotal evidence that can contribute to our understanding of the complex interplay between these distinct yet potentially interrelated conditions. By meticulously examining these real-world cases, researchers and clinicians can identify patterns, trends, and commonalities that indicate a possible association or shared mechanisms [42,49-51].

These reported instances provide a qualitative glimpse into the coexistence of COVID-19 and bacterial endocarditis and act as a foundation for further in-depth investigations. Analyzing these cases may reveal essential details regarding the sequence of events, clinical presentations, and outcomes of patients who experience both conditions simultaneously or sequentially. Such insights can aid in formulating hypotheses, refining research methodologies, and directing prospective studies to explore the potential link between COVID-19 and bacterial endocarditis more systematically [52].

While case studies inherently have generalizability and causal inference limitations, they offer a crucial starting point for generating research hypotheses and guiding subsequent scientific endeavors. By leveraging the collective knowledge derived from these reported instances, researchers can strategically design studies that encompass larger patient populations, incorporate controlled methodologies, and delve into mechanistic investigations. Thus, examining case studies provides anecdotal evidence of a potential relationship and plays a pivotal role in setting the stage for rigorous and comprehensive research that can unravel the complexities of this intriguing interaction between COVID-19 and bacterial endocarditis [53].

Theoretical Mechanisms Linking COVID-19 and Bacterial Endocarditis

The potential connection between COVID-19 and bacterial endocarditis encompasses a complex web of intricate mechanisms that intertwine due to the influence of the viral infection. While definitive causal relationships are still subject to further investigation, several compelling mechanisms warrant thorough consideration due to their potential impact on the development of bacterial endocarditis [54]:

Viral-Induced Endothelial Disruption

SARS-CoV-2, the virus responsible for COVID-19, has demonstrated an unsettling capacity to directly infect endothelial cells, which play a pivotal role in maintaining vascular integrity and function. This viral invasion can lead to significant endothelial dysfunction, disrupting the delicate balance between vasodilation and vasoconstriction. The compromised endothelial barrier, which typically protects against invasive pathogens, could foster an environment conducive to bacterial adherence and colonization. With endothelial cells potentially impaired by the viral assault, the risk of bacterial endocarditis initiation might be heightened, as these compromised sites offer an inviting niche for bacterial biofilm formation on heart valves or endocardial surfaces [55-57].

Immune Modulation and Susceptibility to Bacterial Infection

COVID-19 is renowned for orchestrating intricate immune responses, often involving a surge of pro-inflammatory cytokines colloquially called a cytokine storm. This immune modulation could affect an individual's susceptibility to bacterial infections. Specifically, the perturbation of immune pathways, such as the potential impairment of T-cell function, might compromise the body's innate ability to clear bacterial pathogens effectively. Consequently, individuals grappling with COVID-19-induced immune dysregulation could face heightened vulnerability to bacterial infections, potentially setting the stage for establishing bacterial endocarditis [23].

As we navigate the complexities of these theoretical mechanisms, it is essential to underscore that the relationship between COVID-19 and bacterial endocarditis is likely multifaceted and influenced by many factors. While the mechanisms outlined above provide valuable insights into the potential interplay, they also underscore the need for rigorous research to establish the nature and extent of these connections. Such research endeavors promise to enhance our comprehension of the pathophysiology and pave the way for more effective prevention strategies and targeted therapeutic interventions [58].

Clinical and diagnostic challenges

Differential Diagnosis and Overlapping Symptoms

The convergence of clinical symptoms between COVID-19 and bacterial endocarditis presents a complex diagnostic challenge for clinicians. The shared presentation of symptoms, such as fever, fatigue, and shortness of breath, can lead to confusion and hinder swift and accurate diagnosis. Distinguishing between these two distinct yet potentially co-occurring conditions becomes imperative for guiding effective management strategies. In this section, we delve into the intricacies of differential diagnosis and the nuanced evaluation required to distinguish between COVID-19 and bacterial endocarditis. By understanding the subtle differentiating features and recognizing the potential overlaps, clinicians can make informed decisions that optimize patient care and prevent delays in appropriate interventions [49].

Impact of COVID-19 on Diagnostic Accuracy

The presence of COVID-19 can significantly impact the diagnostic landscape of bacterial endocarditis. The systemic effects of COVID-19, including inflammation, cytokine release, and cardiac involvement, can complicate the interpretation of diagnostic tests commonly used for bacterial endocarditis, such as blood cultures and echocardiography. This section critically examines how the viral infection's influence on the host's physiology and immune response may lead to false positives or negatives in traditional diagnostic markers. Recognizing these potential pitfalls and understanding the altered diagnostic accuracy in the context of COVID-19 is paramount. By mitigating the impact of these challenges on diagnostic confidence, clinicians can ensure appropriate and timely management decisions for patients at risk of both viral and bacterial infections [59].

Strategies for Accurate Diagnosis and Differentiation

The complexity of differentiating between COVID-19 and bacterial endocarditis demands thoughtful and systematic approaches to achieve accurate diagnosis and effective treatment. Leveraging a combination of clinical, laboratory, and imaging data becomes essential in unravelling diagnostic uncertainties. This section explores strategies for enhancing diagnostic precision, including incorporating modified diagnostic criteria, a multidisciplinary clinical approach, and the judicious use of advanced imaging techniques. By integrating these strategies, clinicians can navigate the intricate web of overlapping symptoms and establish a robust framework for identifying the underlying cause, guiding tailored and optimized therapeutic interventions. The pursuit of accurate diagnosis and differentiation contributes to individual patient outcomes. It has broader implications for health systems striving to deliver effective and targeted care amidst the complexities of dual disease burdens [60].

Management and treatment considerations

Current Guidelines for Managing COVID-19-Associated Cardiovascular Complications

The dynamic interplay between COVID-19 and cardiovascular complications underscores the importance of a comprehensive and multidisciplinary approach to patient care. As COVID-19 continues to reveal its intricate impact on the cardiovascular system, it becomes imperative to navigate these complexities guided by up-to-date clinical guidelines. This section delves into the current recommendations for managing COVID-19-associated cardiovascular complications, drawing from the collective expertise of healthcare organizations and research institutions. By synthesizing these guidelines, we aim to provide clinicians with a roadmap for optimizing patient outcomes, enhancing risk assessment, and tailoring therapeutic interventions to address the intricate spectrum of cardiovascular manifestations witnessed in COVID-19 patients [15,30].

Treatment Strategies for Bacterial Endocarditis in the Context of COVID-19

Managing bacterial endocarditis amid the backdrop of COVID-19 introduces unique challenges that demand meticulous consideration of treatment priorities. The convergence of two distinct yet intertwined clinical conditions necessitates a nuanced approach acknowledging potential interactions, synergies, and potential conflicts between therapeutic strategies. This section delves into the intricacies of treating bacterial endocarditis in COVID-19, where therapeutic decisions must be made precisely to ensure optimal patient outcomes. By exploring adaptive treatment approaches, potential challenges, and strategies to harmonize interventions, we strive to equip healthcare professionals with the knowledge to navigate the complex landscape of dual clinical priorities [61].

Antimicrobial Therapy and Potential Drug Interactions

Antimicrobial therapy is the cornerstone of bacterial endocarditis treatment, which assumes an even more critical role when managing patients with concomitant COVID-19. However, the intricate dance of drug interactions, adverse effects, and pharmacokinetic considerations takes center stage in these complex clinical scenarios. This section delves into the multifaceted realm of antimicrobial therapy, investigating the potential interplay between antibiotics and antiviral agents used in both conditions. By scrutinizing potential drug interactions and their implications, we empower healthcare providers to make informed therapeutic choices, ensuring effective bacterial endocarditis management while addressing the intricacies of COVID-19 pharmacotherapy. This holistic approach underscores the paramount importance of patient safety, treatment efficacy, and interdisciplinary collaboration in the face of these intricate medical challenges [62,63].

Future directions and research implications

Areas for Further Investigation

Advancements in our understanding of the intricate interplay between COVID-19 and bacterial endocarditis are of paramount importance, as they hold the potential to influence clinical practice and shape public health policies significantly. This section highlights critical domains warranting intensive investigation, contributing to a more comprehensive grasp of these complex interactions [42]:

Long-term cardiovascular sequelae of COVID-19: The exploration of the enduring cardiovascular repercussions of COVID-19 stands as a critical frontier in research. Efforts should be directed toward unravelling the trajectory of cardiovascular complications in individuals who have recuperated from COVID-19. Understanding the persistence and evolution of these complications can shed light on the potential long-term implications, including their role in predisposing individuals to bacterial endocarditis and other cardiovascular sequelae [7].

Prospective studies on COVID-19 and bacterial endocarditis incidence: Establishing well-designed prospective studies is imperative to decipher the potential influence of COVID-19 on the incidence of bacterial endocarditis. Rigorous epidemiological investigations, incorporating robust methodologies, can provide essential insights into the nuanced relationship between these entities. Such research holds the promise of informing preventive strategies and the potential to guide clinical decision-making in a more evidence-based manner [43].

Therapeutic and Preventive Avenues

Navigating the intricate landscape of COVID-19 and bacterial endocarditis calls for proactive exploration of therapeutic and preventive strategies. This section delves into potential avenues for intervention, aiming to mitigate the multifaceted impact of these clinical interactions [42]:

Vaccine development: Developing and deploying effective vaccines against SARS-CoV-2 represent a pivotal cornerstone in our fight against COVID-19-associated complications. Vaccination strategies extend beyond viral containment, potentially contributing to a broader shield against cardiovascular sequelae. By reducing the risk of viral-induced endothelial dysfunction, vaccines may indirectly modulate susceptibility to bacterial infections, including endocarditis [4,64].

Targeted antiviral and antibacterial therapies: Investigating tailored antiviral therapies specifically designed to combat SARS-CoV-2 could hold the key to mitigating viral-induced endothelial damage and its downstream cardiovascular consequences. Simultaneously, pursuing innovative antibacterial treatments or synergistic therapeutic approaches can potentially enhance the management of bacterial endocarditis in the backdrop of COVID-19. Developing strategies holistically addressing viral and bacterial threats can optimize patient care and improve clinical outcomes [64].

## Conclusions

This review article has delved into the interplay between COVID-19 and bacterial endocarditis, shedding light on the potential pathways and mechanisms underlying the heightened risk of cardiovascular complications in individuals afflicted by both conditions. The convergence of viral-induced endothelial dysfunction, immune dysregulation, and microbial translocation creates a conducive environment for bacterial colonization within the endocardium. The multifaceted nature of these interactions underscores the importance of a holistic approach to patient care, encompassing both viral and bacterial aspects. As we continue to grapple with the aftermath of the COVID-19 pandemic, clinicians and researchers must maintain vigilance in monitoring and addressing cardiovascular health in patients recovering from SARS-CoV-2 infection. Early recognition of potential bacterial endocarditis, particularly in those with preexisting cardiac conditions, can significantly influence patient outcomes. Future research endeavors should focus on elucidating the precise molecular mechanisms driving the observed associations, thus providing a solid foundation for developing targeted therapeutic strategies. Ultimately, exploring the intricate link between COVID-19 and bacterial endocarditis not only enhances our understanding of the disease processes but also highlights human health's dynamic and intricate nature. By unravelling these complex interactions, we can aspire to more effectively mitigate the impact of COVID-19 on cardiovascular health and pave the way for improved clinical management and patient outcomes.
